# Invasive plants facilitated by socioeconomic change harbor vectors of scrub typhus and spotted fever

**DOI:** 10.1371/journal.pntd.0007519

**Published:** 2020-01-21

**Authors:** Chen-Yu Wei, Jen-Kai Wang, Han-Chun Shih, Hsi-Chieh Wang, Chi-Chien Kuo

**Affiliations:** 1 Department of Life Science, National Taiwan Normal University, Taipei, Taiwan; 2 Center for Diagnostics and Vaccine Development, Centers for Disease Control, Ministry of Health and Welfare, Taipei, Taiwan; 3 Institute of Environmental and Occupational Health Sciences, College of Public Health, National Taiwan University, Taipei, Taiwan; University of Cincinnati, UNITED STATES

## Abstract

**Background:**

Ecological determinants of most emerging vector-borne diseases are understudied, particularly for neglected tropical disease. Moreover, although socioeconomic impacts can have significant downstream effects on human risks to vector-borne diseases via a change in land cover, particularly facilitating the invasion of exotic plants, related studies remains very scarce. Scrub typhus and spotted fever are neglected diseases emerging around the globe and are transmitted by chigger mites and ticks infective of *Orientia tsutsugamushi* and *Rickettsia* spp., respectively, with small mammals as the primary hosts of both vectors.

**Methodology/Principal findings:**

We investigated how invasions of the plant *Leucaena leucocephala* caused by widespread abandonment of farmlands driven by industrialization affected abundance of chiggers and ticks in Penghu Island, Taiwan. We determined ectoparasite abundance by trapping small mammals in three types of habitats (invasion site, agricultural field, human residential) every two months for a year. Based on ectoparasite burdens, invasion sites harbored more chiggers and ticks than the other two habitats. Furthermore, hosts maintained higher burdens of both vectors in early winter and burdens of chiggers were more stable across seasons in invasion sites, suggesting that sites with invasive plants could be a temporary refuge for both vectors and might help mitigate the negative influence of unfavorable climate. Infective rates of *O*. *tsutsugamushi* in chiggers and *Rickettsia* in ticks were also consistently not lower in invasion sites. Top soil temperature and relative humidity were similar across the three habitats, but invasion sites contained more of the rat *Rattus losea*, on which chiggers and ticks were more engorged than those from the most commonly trapped species (*Suncus murinus* shrew), indicating that abundance of the host *R*. *losea* instead of microclimate might better determine the abundance of both vectors.

**Conclusions/Significance:**

This study highlights an important but largely neglected issue that socioeconomic change can have unexpected consequences for human health induced particularly by invasive plants, which could become a hotspot for emerging infectious diseases but usually are very hard to be eradicated. In the future, a more comprehensive approach that integrates socio-economics, land use, exotic species, and human health should be considered to fully understand potential emergence of vector-borne diseases.

## Author summary

Understanding how environmental factors, such as land use change, affect vector-borne disease risk helps control and prevent human diseases. However the ecological preferences associated with these vectors of many neglected diseases remains poorly investigated. In this study, we found that abundance of vectors of scrub typhus (chigger mites) and spotted fever (hard ticks), two emerging neglected diseases, on primary hosts were much higher in sites invaded by exotic plants than the other major land cover types in a small island of Taiwan. Additionally, ectoparasite burdens of chigger mites in invasion sites were more stable across seasons, suggesting that plant invasion sites could be a refuge for disease vectors even under unfavorable climatic conditions. Higher abundance of chigger mites and ticks was related to a higher abundance of a superior rodent host instead of a difference in soil microclimate. More significantly, the establishment of these invasive plants has been facilitated by extensive abandonment of farmlands driven by industrialization and rural to urban human migration, thus demonstrating an important but largely overlooked issue that socioeconomic change, when mediated through a change in land cover, can have unexpected downstream effects on emerging neglected tropical diseases.

## Introduction

Many vector-borne diseases are emerging around the globe, but the importance of ecological factors in driving these emergences, such as climate change and land use change, remains largely unconfirmed [1,2], particularly when concerning neglected tropical diseases. There is growing concern that plant invasions can have unexpected consequence for human health, including risks to vector-borne diseases [3]. Limited studies revealed that exotic plants can increase or sometimes decrease abundance of disease vectors. For example, there were more tick vectors of Lyme disease in Japanese barberry invasion sites than in areas dominated by native shrubs [4–7]. Likewise, ehrlichiosis-transmitting ticks were more abundant in sites occupied by invasive Amur honeysuckle than in sites free of it [3]. Invasive plants can also benefit some mosquito species [8–11]. By contrast, exotic plants can reduce the survival of some ticks [12] or result in less preferred oviposition sites for mosquito vectors of La Crosse virus [13].

However, the aforementioned studies were typically conducted during a limited period of the year, without further investigating whether the extent or direction vector survival or abundance in invaded versus non-invaded habitats might vary seasonally. For example, invasive plants might help vectors endure unfavorable weather or season by maintaining more stable climatic conditions under dense vegetative cover or by providing refuges for vertebrates that act as hosts for some disease vectors (such as ticks and some mite species). If these prove to be the case, eradicating invasive plants will become more pressing when these plants can ameliorate the negative effects of extreme weather conditions under further climate change. Furthermore, to better predict human risks to vector-borne diseases after plant invasion, elucidating mechanisms capable of enhancing or suppressing disease vectors is essential, but relevant studies remain very scarce (but see [3,10,12]). Abundance of Acari disease vectors like ticks can be determined by both abiotic and biotic factors since their life cycles include free-living in soils and dependence on vertebrate hosts [3]. Abiotic factors include changes to soil surface microclimate after plant invasions, which can affect the survival of questing ticks [12]. Additionally, invasive plants can lead to increased aggregation of ticks by providing food or cover for their vertebrate hosts [3] that are essential for ticks to lay eggs or molt to the next life stage.

Scrub typhus and spotted fever are neglected diseases that are emerging around the globe [14,15]. Scrub typhus is an acute, potentially lethal febrile disease transmitted by chigger mites (Trombiculidae) infected with the rickettsia *Orientia tsutsugamushi* (OT) and has long been thought confined to Asia and northern Australia [16]. However, this disease has recently been identified in South America (Chile and Peru [17–19]) and Africa (Kenya and Djibouti [20–22]), and is also emerging in some endemic regions, such as China and Korea [23–27]. The life cycle of chigger mites include the egg, larva, nymph, and adult; only the larval stage (chiggers thereafter) is parasitic. Chiggers feed primarily on rodents and are the only stage capable of transmitting OT to humans, while nymphs and adults free live in soil and predate on arthropods [28–30]. Chigger mites are the only reservoir of OT [14,31], with extremely high transstadial (from larva to nymph to adult) and transovarial (from adult to progeny) transmission efficiency [32–33]. Vertebrate hosts only provide sources of food to chiggers but play no role in transmitting OT [14]. Because chigger mites spend >99% of their life in soil [34], other than rodents as the main food resource of parasitic chiggers, soil temperature and moisture are the main determinants of their abundance and distribution [29,30].

Likewise, spotted fever is emerging around the globe and is transmitted primarily by hard ticks (Ixodidae) infected with spotted fever group (SFG) rickettsiae (*Rickettsia* spp.) [35,36]. Similar to chigger mites, life stages of hard ticks include eggs, larvae, nymphs, and adults. However, in contrast to chigger mites, hard ticks can be parasitic throughout all stages of their life cycles, requiring blood meal from vertebrates to molt or lay eggs. Ticks are reservoirs of SFG rickettsiae, which can be transmitted both transstadially and transovarially, and all three parasitic stages are capable of infecting humans with SFG rickettsiae. Although it is less clear whether vertebrate hosts also serve as reservoirs of these pathogens [37]. Like chigger mites, hard ticks spend a great proportion of their life cycle on the ground (>90%, [38]), so their population is also affected by soil temperature and humidity [38–40].

Meanwhile, socioeconomic-driven change can have impacts on land use and land cover, which includes invasions of exotic plants. Even though farming continues to dramatically transform Earth’s landscapes [41], global abandonment of agricultural fields has also increased considerably since the 1950s [42]. Abandonment usually occurs in remote, marginal agricultural lands, where soil fertility is poor and yields are low [43–46]. Socio-economic factors that have led to the depopulation of rural areas and subsequent abandonment of fields [47] include industrialization, rural-urban migration, and urbanization [43–46,48,49]. These abandoned old fields, particularly degraded lands with strong cultivation legacy, are typically dominated by highly competitive invasive plants that can impede the recovery of native plants [42,45,48,50].

**Fig 1:**
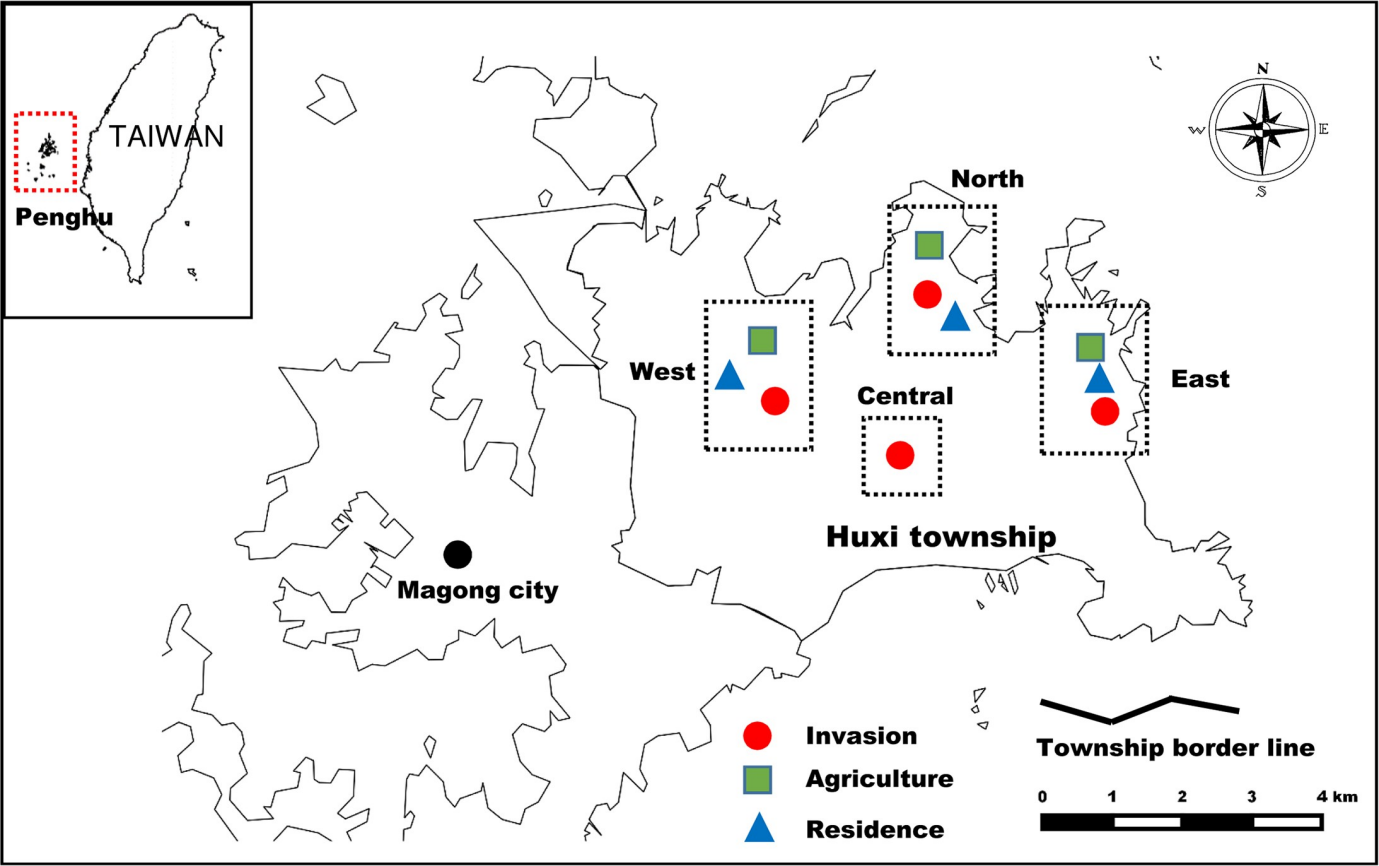
Study sites in Huxi township of Penghu Islands. The maps were created by the authors with QGIS 2.12.2-Lyon by QGIS Development Team.

**Fig 2:**
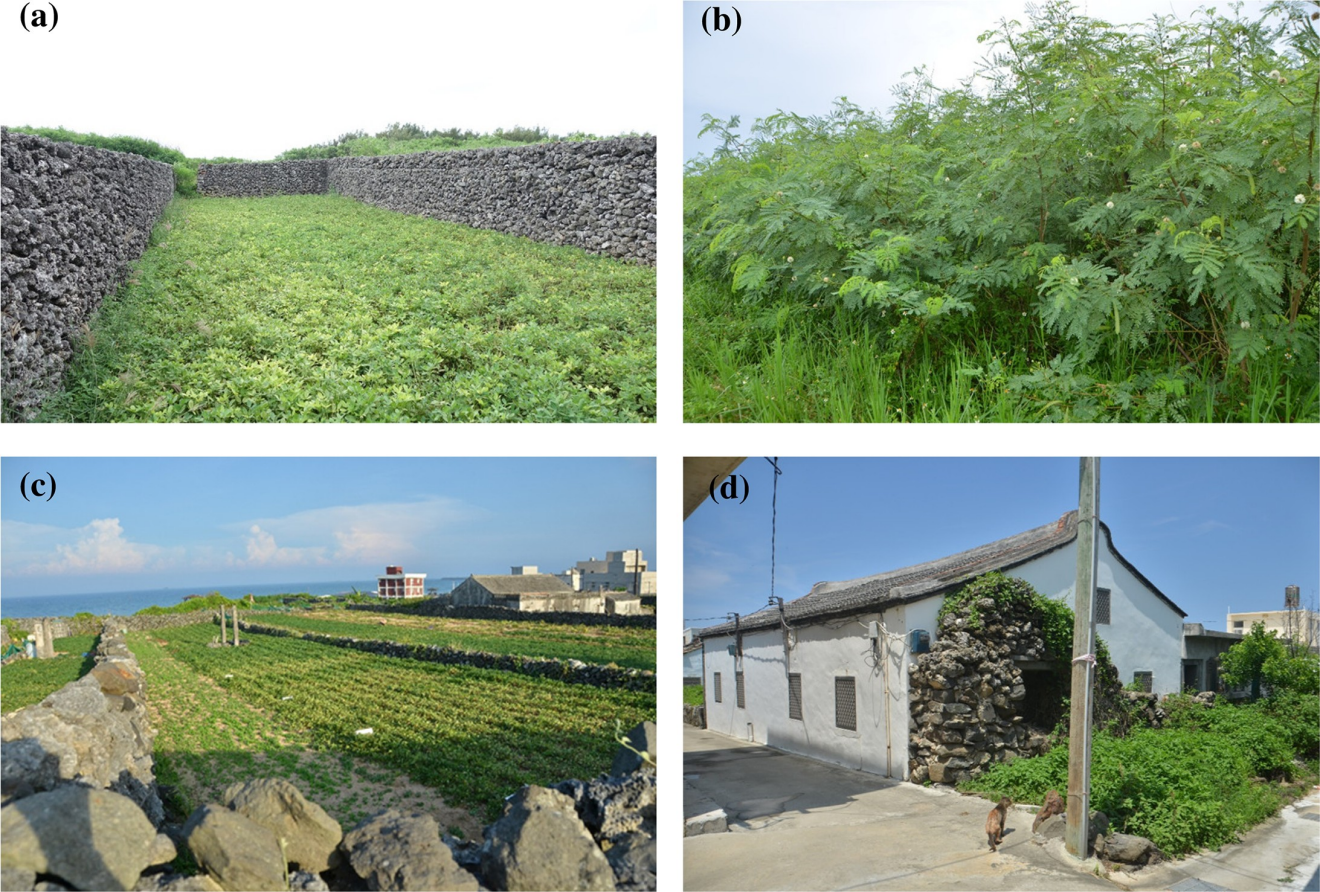
Habitats in Penghu Islands. (a) Farmers typically use coral stones to build walls for fending off strong wind during the winter; (b) *Leucaena leucocephala* invasion sites; (c) agricultural fields; (d) human residential sites.

**Fig 3:**
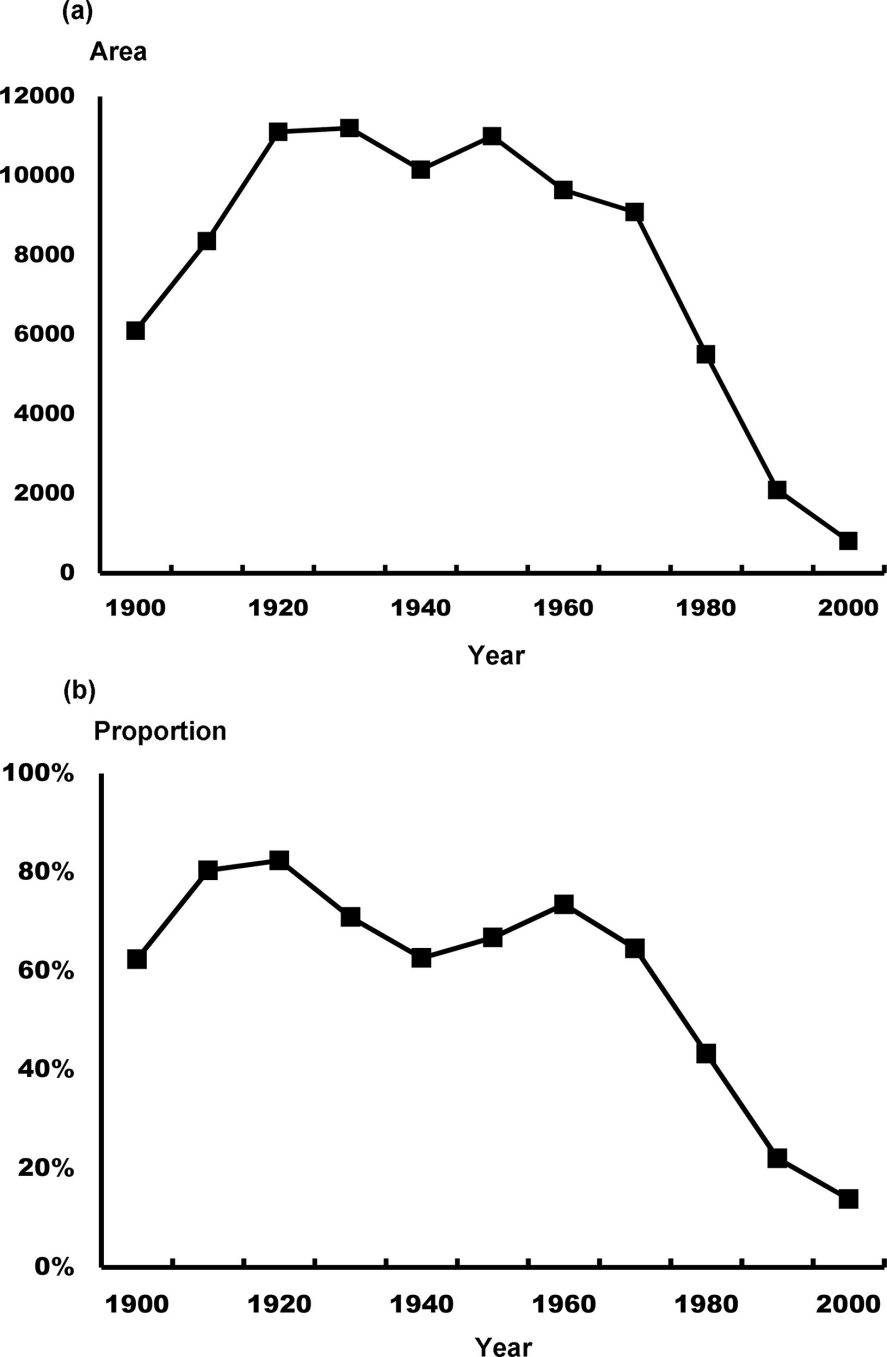
Annual variation in (a) area of cultivated lands (ha) and (b) proportion of workers that were farmers in Penghu Islands.

The Penghu Islands, previously known as the Pescadores Islands, are located in the Taiwan Strait (Fig 1) and are comprised of 90 subtropical and tropical islands, with the largest island covering an area of 65 km^2^. The climate in Penghu is characterized by hot summers and dry, windy winters [51]; farmlands are usually surrounded by walls made of coral stones to fend off strong winds (Fig 2A). Moreover, due to the small size of the islands, exposure to sea water is extensive, particularly during the windy winters, which has led to high soil salinity. Unfavorable climatic conditions and poor soil fertility greatly limit agricultural productivity in Penghu. Furthermore, when industrialization took off in Taiwan in the early 1970s, Penghu saw a sharp decline in agricultural activity; more specifically, fewer cultivated lands and agricultural workers (Fig 3, adapted from [51]). A major outcome is that most agricultural fields in Penghu have been abandoned. For example, as of 2016, about 70% of workers were in the service sector [52], and in 2015, 75% of farmlands were abandoned, the highest among all counties in Taiwan; around 45% higher than the next highest county [53]. In addition, as of 2012, more than half of all area in Penghu Islands were comprised of abandoned fields (51.9%), followed by artificial facilities (11.3%) and agricultural fields (10.2%) [54]. These abandoned fields are invaded almost exclusively by a nitrogen-fixing legume, the exotic white popinac *Leucaena leucocephala* (family Fabaceae), which is among 100 of the world’s worst invasive alien species listed by IUCN Invasive Species Specialist Group [55]. *L*. *leucocephala*, native to Central America, has been introduced worldwide as firewood or fodder plants and can become highly invasive in disturbed regions with dry and poor soil. Additionally, they can prevent native vegetation recovery by forming dense thickets that are very difficult to eradicate (Global Invasive Species Database, IUCN, http://www.iucngisd.org/, accessed October 17, 2018). In Penghu, the density of *L*. *leucocephala* can reach 30,000 to 50,000 stands per hectare [56] and eradicating *L*. *leucocephala* has been a priority for the local government. Moreover, land untended after the removal of *L*. *leucocephala* will quickly be reclaimed by the invasive species due to its high soil seed density (as many as 2,000 seeds per square meter; [57]). This demonstrates that land use should be a stronger regulating factor of invasions of *L*. *leucocephala* compared to long-term climate change.

Penghu Islands, at the same time, is a hotspot of scrub typhus, with the highest number of documented human cases of scrub typhus among all counties in Taiwan for the past ten years (2008–2017, Taiwan Centers for Disease Control, https://nidss.cdc.gov.tw/, accessed October 17, 2018). Despite the U.S. Naval Medical Research Unit Two studying scrub typhus cases in Penghu in the 1960s and 1970s [58–64], these studies did not focus on ecological factors, such as assessing habitat differences in chigger vectors and the significance of invasive plants. Likewise, SFG rickettsiae have been detected in hard ticks and small mammals in Penghu [65–66], but without investigating the importance of habitat type in sustaining disease vectors.

Although socioeconomic change can have significant downstream effects on human risks to vector-borne diseases via changes in land use and vegetative community, these types of studies remain very limited. Here, we investigated (1) whether the invasion of *L*. *leucocephala*, facilitated by socioeconomic change, creates better habitats for chiggers and ticks by comparing loads of both vectors on small mammal hosts among habitats. A comparison among habitats was also implemented across seasons to assess if invasive plants help sustain vector populations under unfavorable climatic conditions. (2) Furthermore, we evaluated the importance of abiotic versus biotic factors in determining differential vector populations between habitats. Here, abiotic factors were defined as top soil temperature and relative humidity while biotic factors included abundance and species composition of small mammal hosts without considering individual host characteristics, such as sex and body mass. (3) Lastly, engorgement degree or feeding success of chiggers and ticks can vary depending on host species [67,68], and a heavily parasitized host species could instead act as ecological traps and lower vector population [67]. This stresses that a host species with high vector load might not necessarily represent a good host. Therefore, we also investigated the engorgement degree of vectors between different host species in order to uncover whether preserving some host species may lower vector populations and reduce disease risks to humans.

## Materials and methods

### Ethical statement

All animal handling procedures were approved by the National Taiwan Normal University Animal Care and Use Advisory Committee (permit number NTNU-104016), which adheres to Guideline for the Care and Use of Laboratory Animals established by the Council of Agriculture, Taiwan.

### Study sites, small mammal trapping, and ectoparasite collection

This study was conducted in Huxi Township, Penghu, Taiwan (Fig 1), where more than half of all Penghu scrub typhus human cases have occurred during the last ten years (2008–2017, Taiwan Centers for Disease Control, https://nidss.cdc.gov.tw/, accessed October 17, 2018). Three types of habitats were compared: areas with *L*. *leucocephala* invasions, agricultural fields, and outdoor human residential areas (Fig 2B–2D). In invasion sites, at least 90% of the land was covered with *L*. *leucocephala*. Agricultural fields were planted with peanuts; intruding weeds, including *L*. *leucocephala*, were routinely removed by farmers. In human residential sites, grounds (including houses, roads, and squares) were paved, and plants of the family Asteraceae and Gramineae were sparsely distributed; a few *L*. *leucocephala* stands occasionally occurred. A total of ten sites were surveyed in our study, including four *L*. *leucocephala* invasion sites, three agricultural fields, and three human residential sites in four different parts (east, west, north, and central) of Huxi (Fig 1). Central Huxi is covered almost exclusively with invasive *L*. *leucocephala*; in order to incorporate this region to obtain a more comprehensive understanding of Huxi, we set up one extra invasion site in central Huxi but were unable to find suitable study sites for agricultural cultivation and human residential sites. *L*. *leucocephala* invasion and human residential sites were larger than 100 ha in size (ranges: 110–160 ha, 100–115 ha for *L*. *leucocephala* invasion and human residential sites, respectively) but the largest agricultural fields we could find and survey were around 10 ha (9.5–12 ha). From December 2016 to October 2017, small mammal traps were set up in each of these ten sites every two months. A total of 30 Sherman small mammal traps (26.5 × 10.0 × 8.5 cm) were deployed in each site. Traps, baited with sweet potatoes smeared with peanut butter, were deployed along a single transect line at 10-m intervals. During each sampling session, traps were open for three consecutive nights and all ten sites were surveyed within 10 days. A given site from each of the three habitat types were always sampled at the same time to avoid any potential temporal variation.

Trapped small mammals, including shrews and rodents, were examined to determine species, sex, body weight, and body length. We checked for presence of ectoparasites by thoroughly examining the whole body of the animal with the naked eye. Skins with attached chiggers, largely in the ears, were removed from host animals with tweezers and placed in vials. After 2–3 days when the chiggers had released from the host skin we added 100% ethanol to the vials in order to preserve intact oral parts for later species identification. Ticks were carefully collected with tweezers and preserved in 100% ethanol. All chiggers and ticks that we collected were stored in a -20°C freezer for subsequent molecular determination. In this study, we did not use metal ear tags for identification of small mammals because the ears of some of our target host species, including *Suncus murinus* and *Mus musculus*, are too small to be fit with ear tags. Additionally, tags can be lost during agonistic encounters, and most importantly, ear tags can increase infestation rates of ticks [69]. Therefore, large rodent species, including *R*. *losea* and *Rattus norvegicus*, were each implanted with a radio-frequency identification chip (Watron Technology Corporation, Hsinchu, Taiwan) for individual identification before release. Smaller species (*S*. *murinus* and *M*. *musculus*) were unable to be permanently marked without difficulty, so were released without being marked.

### Species identification and engorgement degree of chiggers and ticks

Chiggers were slide-mounted in Berlese fluid (Asco Laboratories Ltd, Manchester, U.K.) and morphologically identified to a species level under a compound microscope following [70]. Ticks were morphologically identified to the species level and life stage (larva, nymph, male adult, female adult) under a dissecting microscope following published keys [71]. When species could not be recognized morphologically, a molecular identification was completed by comparing 12S rDNA and 16S rDNA to known species following [72,73]. All ticks were identified, while due to the very large number of chiggers, only a portion of chiggers collected from each individual host (>25% of chiggers from each individual host) were randomly selected and examined.

Degree of engorgement of chiggers and ticks was compared among host species. Engorgement degree of a chigger was determined by the increase in idiosoma area relative to the one with the smallest idiosome area, which we calculated by using the ellipse equation [68]. Engorgement degree of chiggers was averaged within each host individual before subsequent comparison among host species. Engorgement degree of ticks was divided into three categories: non-engorged, half-engorged, and fully engorged. Unlike chiggers, interspecific comparisons of ticks were based on individual ticks irrespective of whether ticks were collected from the same host individual.

When collecting chiggers from captive hosts, chiggers that naturally complete feeding, release from the host, and drop to the water pan underneath the host-housing cage are very difficult to find when the minute chiggers are mixed with feces and food remains discarded by the caged host. Contrarily, although collecting host-releasing ticks in this manner is possible, engorged chiggers drop to the water pan at the same time as ticks do may nevertheless be overlooked as aforementioned. In this study, host quality was therefore represented by engorgement degree of vectors immediately after they were collected and preserved in ethanol instead of allowing them to finish engorging or molting. The hosts were concomitantly infested with vectors in different stages of engorgement; any observed discrepancy in engorgement degree will not reflect intrinsic difference in host quality only when vectors collected from one host species happened to be always in their late stage of engorgement (thus more fully engorged) while vectors from the other host species always in their early stage of engorgement, which is unlikely. Therefore, when a large difference in engorgement degree of chiggers and ticks was observed among host species, this demonstrates that while what we measured might not be the best indicator (i.e. by allowing them to finish engorging or molting), it is nonetheless useful to discern any large discrepancy in host quality.

### Detection of OT in chiggers and *Rickettsia* spp. in ticks

Due to the small size of chiggers, a sum of 30 chiggers from the same host individual was pooled for detection of OT with nested polymerase chain reaction (PCR) following [74], which targeted the well conserved 56-kDa type specific antigen located on the OT outer membrane. Laboratory OT strains and nuclease free water were used as positive and negative controls, respectively. Ticks were individually assayed for presence of *Rickettsia* spp. with nested PCR following [66], which targeted the 120- to 135-kDa surface antigen (*ompB*) and citrate synthase (*gltA*). Laboratory *R*. *rickettsii* antigen and nuclease free water were used as positive and negative controls, respectively. In this study, ticks were assayed for presence of *Rickettsia* without further sequencing to identify the species, which could be endosymbionts of ticks and thus non-pathogenic to humans. Nevertheless, our previous studies identified agents of tick-borne spotted fever, including *Rickettsia conorii* and *R*. *rickettsii*, in ticks and small mammals in Penghu Islands [65, 66], indicating that *Rickettsia* spp. capable of causing spotted fever are present in Penghu.

### Top soil temperature and relative humidity

Temperature and relative humidity of the top soil were recorded from December 2016 to October 2017 by placing a data logger (WatchDog, Spectrum Technologies Inc., East Plainfield, Illinois) on the ground at each of the 10 study sites. The data loggers were deployed under dense *L*. *leucocephala* stands, in the open field, and by the outside, south-facing walls of buildings in invasion sites, agricultural fields, and human residence sites, respectively, aiming to measure representative microclimate for each habitat type although such method (with only one point measure) might still fail to record the full spectrum of microclimate variation. Measurements were recorded at an interval of 30 minutes.

### Statistical analyses

Since *S*. *murinus* and *M*. *musculus* were not individually marked, only results from the 1^st^ day of capture in each three-day trapping session were used to calculate capture rate (unique individuals/trap-nights) and ectoparasite load (number of all ectoparasites/number of all host individuals). Including the results of 2^nd^ and 3^rd^ trapping day might overestimate capture success (when host individuals were recaptured) and underestimate ectoparasite loads (when chiggers and ticks had been previously removed from recaptured hosts). Additionally, only the results from the 1^st^ trapping day were used to compare host species composition among habitats. However, when tallying the total number of ectoparasites in each site, results from all three days were included. We figured that including even recaptured, unmarked host individuals of *S*. *murinus* and *M*. *musculus* would only slightly increase total ectoparasite numbers as ectoparasites had already been removed from these host individuals the previous one or two days. Although there was chances that recaptured *S*. *murinus* and *M*. *musculus* could include those few individuals more prone to both parasite infestation (the 80/20 rule, 20% of hosts burdened with 80% of parasites, [75]) and capture (“trap happy”), therefore considerably raising the total parasite number, this did not happen in this study for two reasons. Firstly, when skins of hosts were removed along with attached chiggers, lesions left on the animals have rendered recaptured individuals in the same 3-day trapping session recognizable (for unmarked *S*. *murinus* and *M*. *musculus*), particularly those with high parasite loads (also those individuals’ recaptures would be more likely to greatly increase the total parasite number if the 80/20 rule holds). However, those recaptured individuals were always lightly re-infested with chiggers (<5 chiggers) and ticks (<2 ticks). Secondly, the fact that there were few chiggers (<10) and ticks (<2) on marked recaptured *R*. *losea*, which was also the species with the highest chigger and tick loads and thus the most competent host (see Results), demonstrated that the infestation pressure of both vectors in our study sites was low at least during the 1- to 2-day period.

### Number and species composition of small mammal hosts across habitats, and engorgement degree and loads of vectors across host species

When comparing engorgement degree of chiggers among host species and number of small mammal captures among habitat types, normality and homogeneity of variance were confirmed with Shapiro–Wilk and Levene tests, respectively. Data were transformed when necessary and if homogeneity of variance cannot be fulfilled even after transformation, Welch’s ANOVA was implemented followed by a Games-Howell post hoc test. When comparing engorgement degree of ticks among host species, whether composition of host species varied among habitats, as well as whether host species varied in their relative importance among habitats in hosting ticks (e.g. ticks might be mostly found on species “A” in habitat 1 but on species “B” in habitat 2), Fisher-Freeman-Halton's tests with 100,000 Monte Carlo permutations were implemented, and if significant, followed by pair-wise Fisher-Freeman-Halton's test with Bonferroni correction. When investigating whether host species varied in their relative importance among habitats in hosting chiggers, Pearson chi-square test was applied. Loads of chiggers and ticks were compared among host species with negative binomial generalized linear mixed models (GLMM) to account for overdispersion of data, as well as controlling for the influence of region, habitat, month, habitat*month (defined as fixed factors), and site (random factor) on loads of ectoparasites. Significant differences were evaluated based on the 95% Wald confidence interval.

### Host capture rate and vector load abundance across regions, habitats, and months

We investigated difference in total number of chiggers and ticks collected from hosts, as well as variation in *R*. *losea* capture rate between regions, habitats, and months with generalized estimating equations (GEE) using a negative binomial log link function, with site as the subject, and each bi-monthly sampling as a repeated measures within the site (ten sites, each with six sampling sessions, so overall 60 samples). Region, habitat, month, and habitat*month were the fixed factors, and significance of difference was determined based on 95% Wald confidence interval of estimated marginal means. The structure of the correlation matrix was selected based on the lowest quasi-likelihood under independence model criterion value. To ensure reliability of the Hessian matrix, the dependent variable (chigger, tick, or *R*. *losea* capture rate) was set as 0.001 when the original value was zero. We ran a GEE model with a normal distribution function when comparing *S*. *murinus* capture rates. Capture rate of marked *R*. *norvegicus* was not compared in this study due to the very few captures of this species. We also calculated the coefficient of variation (CV = standard deviation divided by mean) for chigger and tick abundance across months for each habitat type, represented by the mean of ectoparasite abundance in each site of the same habitat. For example, the abundance of chiggers in *L*. *leucocephala* invasion sites in December 2016 was represented by the average of the chigger abundance of the four *L*. *leucocephala* invasion study sites surveyed in that month.

### Prevalence of OT and *Rickettsia* across host species and habitats

When comparing prevalence of *Rickettsia* presence in vectors collected from different host species and habitats, the more robust bootstrapped logistic regression was applied [76] instead of the conventional logistic regression that could cause biased results when a sample size is small [77]; a 95% confidence interval was estimated with 10,000 permutations. We followed [78] in estimating mean and 95% confidence interval (CI) of individual-level (per chigger) infection prevalence of OT in chiggers with a frequentist approach assuming perfect test, with confidence intervals calculated based on binomial theory.

### Effect size, data presentation, and statistical software

Effect size (mean and 95% CI) was estimated with non-parametric Cliff’s delta δ instead of conventional Cohen’s d or Hedges’ g to accommodate non-normality and heterogeneity in our data; δ quantifies the amount of difference between two groups and ranges from -1.0 to 1.0, with effect size increasing with the value of δ (δ = 0 representing complete overlap in distributions of two groups while δ = 1 or -1 standing for absence of overlap in distributions) [79]. Data are given as the mean ± 1 standard error (SE). Prevalence of OT was calculated with EpiTools Epidemiological Calculators [80], and effect size estimated with the package “effsize” in R [81]. The other statistical procedures were performed in SPSS Statistics version 19.0 (IBM Corp.).

## Results

### Small mammal composition and chigger and tick infestations

A total of 1,323 small mammals of four species were captured out of a sampling effort of 5,400 trap-nights. The *S*. *murinus* shrew was the most abundant (capture rate = 0.207 individuals/trap-nights), followed by the rodents *M*. *musculus* (0.067), *R*. *losea* (0.029), and *R*. *norvegicus* (0.001).

A sum of 40,799 chiggers were collected, primarily from *R*. *losea* (77.4% of total), and to a less extent from *S*. *murinus* (18.1%), *M*. *musculus* (3.0%), and *R*. *norvegicus* (1.5%). Chigger load (number of chiggers/number of host individuals) was significantly higher on *R*. *losea* (185.7±32.4 chiggers, mean ± 1SE, n = 53) than on *S*. *murinus* (8.4±1.9, n = 373), which in turn was higher than *M*. *musculus* (2.9±1.2, n = 120) (negative binomial GLMM, all *p* < 0.05). Only one *R*. *norvegicus* was captured during the 1^st^ trapping day, with a chigger load of one.

A total of 1,042 ticks were collected, including 484 larvae (46.4%), 286 nymphs (27.4%), and 272 adults (26.1%). These were mostly collected from *S*. *murinus* (69.5% of total), followed by *R*. *losea* (25.3%), *M*. *musculus* (4.8%), and *R*. *norvegicus* (0.4%). Tick load was significantly higher on *S*. *murinus* (1.6±0.8, n = 373) and *R*. *losea* (1.3±0.4, n = 53) compared to *M*. *musculus* (0.1±0.04, n = 120) (negative binomial GLMM, both *p* < 0.05) while there was no difference between the first two species (*p* > 0.05). Tick load of the single *R*. *norvegicus* captured during the 1^st^ trapping day was zero.

### Species identification and engorgement degree of chiggers and ticks

**Fig 4:**
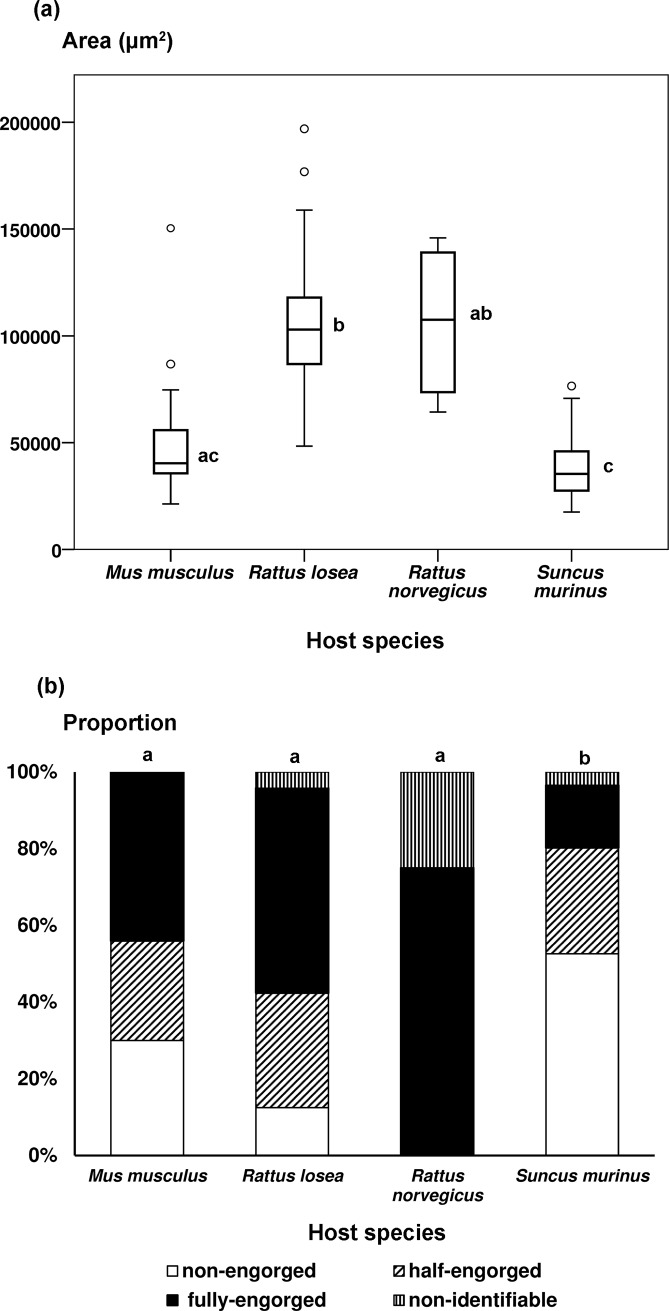
Engorgement degree of (a) chiggers and (b) ticks collected from different host species in Penghu Islands from December 2016 to October 2017. Different letters represent significant difference.

A total of 10,478 chiggers were slide-mounted for species identification, including 1,035 chiggers (9.9% of total) that could not be reliably identified due to inadequate preparation of the specimens. The other 9,443 were successfully identified, which include 324 chiggers from 25 *M*. *musculus*, 7,376 chiggers from 109 *R*. *losea*, 148 chiggers from four *R*. *norvegicus*, and 1,595 chiggers from 66 *S*. *murinus*. All chiggers were identified as *Leptotrombidium deliense*. Engorgement degree varied among host species (Welch’s ANOVA, F_3, 13.3_ = 153.4, *p* < .001), with chiggers on *R*. *losea* (10.4±0.2 x10^4^ μm^2^) more engorged than those on *M*. *musculus* (4.9±0.5 x10^4^ μm^2^) and *S*. *murinus* (3.8±0.2 x10^4^ μm^2^) (Games-Howell test, both *p* < 0.001) (Cliff’s delta δ = 0.89 (95% CI: 0.62–0.97), 0.99 (95% CI: 0.97–1.00, respectively). Engorgement degree on *R*. *norvegicus* (10.6±1.9 x10^4^ μm^2^) was similar as those on *R*. *losea* and *M*. *musculus* (both *p* > 0.05) (δ = -0.02 (95% CI: -0.70–0.68), 0.84 (95% CI: 0.52–0.95), but was larger than those on *S*. *murinus* (*p* < 0.05) (δ = 0.95; 95% CI: 0.96–0.99) (Fig 4A).

The 1,042 collected ticks were comprised predominantly (99.1%) of *Ixodes granulatus*; the other small proportion (0.9%) was *Amblyomma testudinarium*. More than half of the ticks (52.6%) collected from *S*. *murinus* were non-engorged, with only 16.3% fully engorged. On the contrary, 53.4% of ticks collected from *R*. *losea* were fully engorged and only 12.5% were non-engorged (Fig 4B). Engorgement degree of ticks varied among the four host species (Fisher-Freeman-Halton's test, *p* < 0.001), with *S*. *murinus* differing from the other three species (all *p* < 0.05, after Bonferroni correction), while there was no difference among *R*. *losea*, *M*. *musculus*, and *R*. *norvegicus* (all *p* > 0.05) (Fig 4B).

### Variation in *R*. *losea* and *S*. *murinus* capture rate across regions, habitats, and months

**Fig 5:**
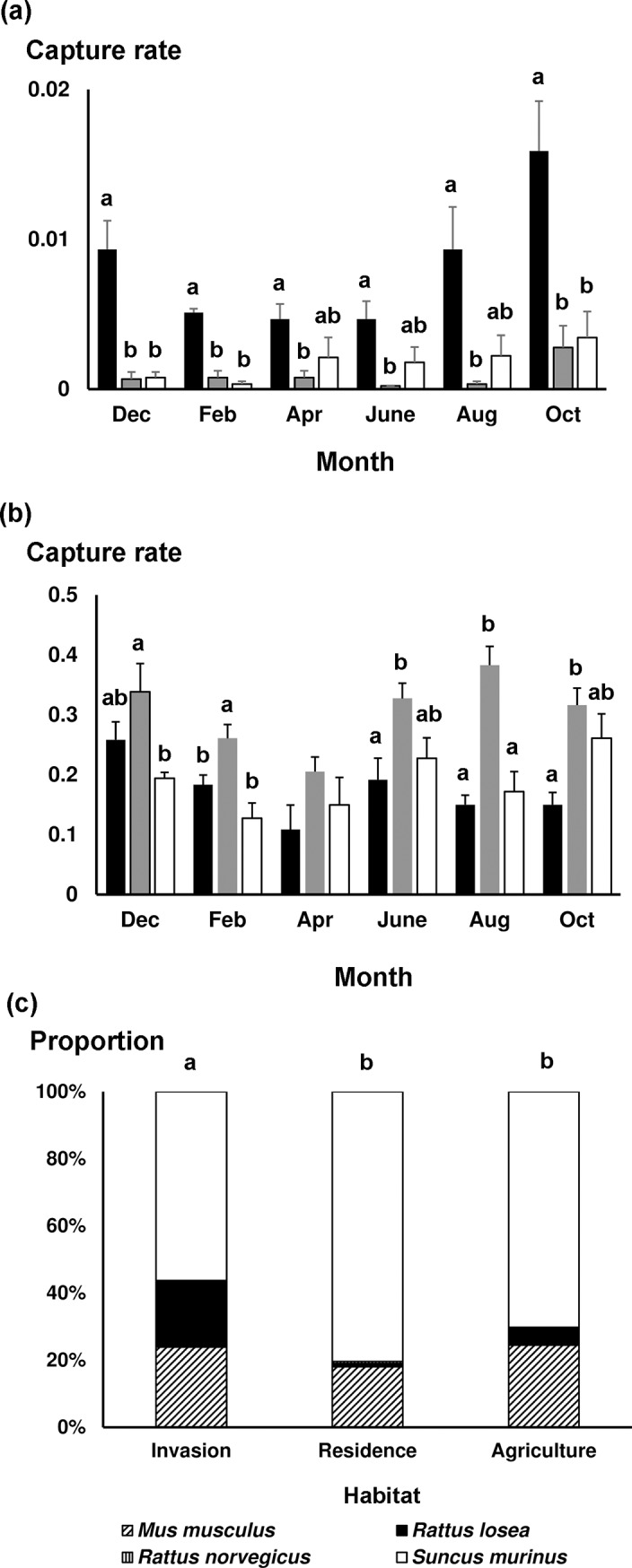
Abundance of small mammals in different habitats in Penghu Islands from December 2016 to October 2017. (a) capture rate of *Rattus losea*; (b) capture rate of *Suncus murinus*; (c) small mammal community composition. Different letters represent significant difference. For (a), (b), statistical comparisons among habitats were implemented only within sampled months and letters were denoted only when significant difference was found. Black: *Leucaena leucocephala* invasion site; grey: human residential site; white: agricultural fields.

Capture rate of *R*. *losea* varied among habitat and month (GEE, both *p* < 0.001), but not region (*p* > 0.05). We also found there was an interaction between habitat and month (*p* < 0.001). Capture rates of *R*. *losea* were higher in invasion sites than both agricultural fields and human residential sites for each month, especially in December (δ = 1 (95% CI: 0.43–1), 0.92 (95% CI: 0.43–0.99)), February (both δ = 1 (95% CI: 0.43–1)), and October (both δ = 1 (95% CI: 0.43–1)) (all *p* < 0.05; Fig 5A). On the other hand, the capture rate of *S*. *murinus* varied among region, habitat, and month (all *p* < 0.001), and there was an interaction between habitat and month (*p* < 0.001). Both eastern and central regions harbored more *S*. *murinus* than western and north regions (all *p* < 0.05). Human residential sites contained more *S*. *murinus* than both invasion sites and agricultural fields for each month; with significance in February (δ = 0.5 (95% CI: -0.52–0.93), 0.78 (95% CI: -0.19–0.98)) and August (δ = 1 (95% CI: 0.43–1), δ = 1 (95% CI: 0.14–1)) (all *p* < 0.05; Fig 5B).

There was no significant difference in number of small mammal captures between invasion (mean = 55.5±4.9 individuals per site), agricultural field (43.7±6.3), and human residential sites (64.7±10.1) (Welch’s ANOVA, F_2, 4.0_ = 1.6, *p* > .05). However, species composition varied among the three habitats (Fisher-Freeman-Halton's test, *p* < 0.001), with invasion sites differing significantly from both human residential sites and agricultural fields (both *p* < 0.05, after Bonferroni correction), while there was no difference between the latter two habitat types (*p* > 0.05). In human residential sites, small mammals were comprised mainly of *S*. *murinus* (80.4% of all hosts), followed by *M*. *musculus* (18.0%) and *R*. *losea* (1.0%). This pattern was similar in the agricultural fields where *S*. *murinus*, *M*. *musculus*, and *R*. *losea* accounted for 70.2%, 24.4%, and 5.3% of total captures, respectively. In comparison, in invasion sites, while *S*. *murinus* was still the most common species (56.3%), about one-fifth (19.8%) of hosts were comprised of *R*. *losea* (Fig 5C).

### Variation in chigger load abundance across regions, habitats, and months

**Fig 6:**
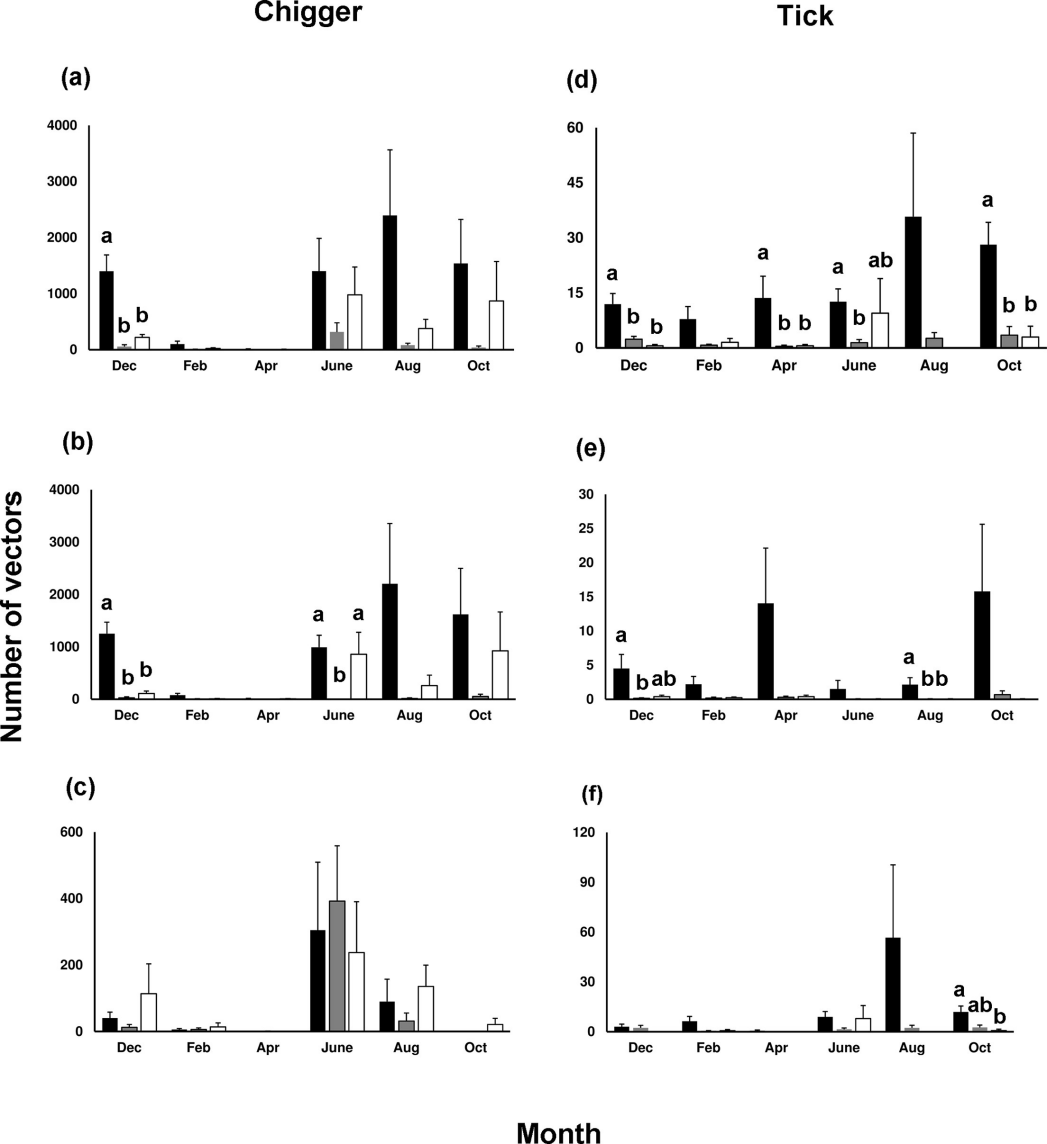
Number of vectors collected from mammal hosts per study site in different habitats and months in Penghu Islands from December 2016 to October 2017. Chiggers collected from (a) all mammals combined; (b) *Rattus losea*; (c) *Suncus murinus*. Ticks collected from (d) all mammals combined; (e) *Rattus losea*; (f) *Suncus murinus*. Different letters represent significant difference; statistical comparisons among habitats were implemented only within months and letters were denoted only when significant difference was found. Black: *Leucaena leucocephala* invasion site; grey: human residential site; white: agricultural fields.

The sum of chiggers collected from all mammal hosts or specifically from *R*. *losea* both varied among region, habitat type, and month (GEE, all *p* < 0.001), and there was an interaction between habitat and month (both *p* < 0.001). There were more chiggers in the eastern region than the other parts of the study area (all *p* < 0.05; S1A and S1B Fig). The number of chiggers collected from hosts in invasion sites were higher than both agricultural fields and human residential sites across all months; with significance in December (all mammal hosts: δ = 0.92 (95% CI: 0.43–0.99), 1 (95% CI: 0.43–1); *R*. *losea*: δ = 0.83 (95% CI: 0.06–0.98), 1 (95% CI: 0.43–1)) (all *p* < 0.05; Fig 6A and 6B). The coefficient of variation (CV) in chigger abundance from hosts across months was lower in invasion sites (CV = 0.79, 0.79; all mammals, only from *R*. *losea*, respectively) than in agricultural fields (1.05, 1.01) and human residence sites (1.60, 1.61).

On the other hand, the number of chiggers collected solely from *S*. *murinus* differed between region and month (both *p* < 0.001) but not between habitat (*p* > 0.05), and there was an interaction between habitat and month (*p* < 0.001). There were significantly more chiggers in the eastern region than the north and west regions (both *p* < 0.05) but not the central (*p* > 0.05; S1C Fig). There were no significant differences among the three habitats within the same month (all *p* > 0.05) (Fig 6C), although the number of chiggers collected were significantly higher in human residential sites in June than the other two habitats in February, April, and October. The CV value was lower in agricultural fields (CV = 1.30) than in invasion sites (1.61) and human residence sites (2.00).

Within the *L*. *leucocephala* invasion sites and agricultural fields, chiggers were collected primarily from *R*. *losea* (89.8%, 74.3%; respectively), whereas in the human residential sites, chiggers were chiefly retrieved from *S*. *murinus* (83.7%). We found that hosts varied in their relative contribution to feeding the chiggers among the three habitats (chi-square test, *χ*^2^ = 16917.7, *p* < 0.001).

### Variation in tick load abundance across regions, habitats, and months

Ticks collected from all mammal hosts, specifically from *R*. *losea* or specifically from *S*. *murinus* all varied among region, habitat, and month (GEE, all *p* < 0.001), and there was an interaction between habitat and month (*p* < 0.001). There were significantly fewer ticks collected in the eastern region than the other regions (all *p* < 0.05; S1D–S1F Fig). Similar to chiggers, a higher number of ticks were collected in invasion sites compared to both agricultural fields and human residential sites in all months (Fig 6D–6F); with significant differences in December (δ = 0.83 (95% CI: 0.06–0.98), 0.67 (95% CI: -0.40–0.97)), April (both δ = 0.83 (95% CI: 0.06–0.98)) and October (δ = 0.83 (95% CI: -0.50–0.99), 0.75 (95% CI: -0.40–0.98)) for all mammals (all *p* < 0.05, Fig 6D), and in August for *R*. *losea* only (both δ = 0.75 (95% CI: -0.40–0.98)) (all *p* < 0.05; Fig 6E), but mostly without significant difference for ticks collected solely from *S*. *murinus* (Fig 6F). The CV value for tick abundance across months was lower in agricultural fields (CV = 0.54) than in human residential (0.62) and invasion sites (0.80) for all mammals, lower in invasion sites (0.73) than in agricultural fields (1.18) and residential sites (1.03) for *R*. *losea* only, and lower in residential sites (CV = 0.71) than in invasion sites (1.22) and agricultural fields (1.40) for *S*. *murinus* only.

Within the *L*. *leucocephala* invasion and human residential sites, ticks were collected principally from *S*. *murinus* (69.8%, 73.6%; respectively), while in the agricultural fields, ticks were collected equally from *R*. *losea* and *S*. *murinus* (both 45.5%). Hosts varied in their relative importance for feeding ticks among the three habitats (Fisher-Freeman-Halton's test, *p* < 0.001).

### Prevalence of OT in chiggers and *Rickettsia* in ticks

A total of 154 pools of *L*. *deliense* chiggers were assayed for OT infections, with a mean individual-level (per chigger) prevalence of 0.80% (95% CI: 0.55–1.12%). Prevalence was higher when chiggers were collected from *R*. *losea* (1.12%, 0.75–1.59%, 105 pools—representing mean, 95% CI of prevalence, and number of pools tested) than from *S*. *murinus* (0.18%, 0.02–0.63%, 39 pools) and *M*. *musculus* (0%, 7 pools), whereas chiggers from *R*. *norvegicus* (1.34%, 0.03–7.57%, 3 pools) were the same as those from *R*. *losea* and *S*. *murinus*, but higher than *M*. *musculus*. For chiggers from *R*. *losea*, prevalence of OT infection was higher when *R*. *losea* was trapped in *L*. *leucocephala* invasion (1.38%, 0.91–2.00%, 82 pools) and human residential sites (1.11%, 0.12–4.04%, 7 pools) than in agricultural fields (0%, 16 pools), but was similar between the first two habitats. For chiggers from *S*. *murinus*, the prevalence was higher in invasion sites (0.51%, 0.01–2.84%, 7 pools) and agricultural fields (0.27%, 0.01–1.48%, 13 pools) than in human residential sites (0%, 19 pools), while there was no difference between the first two habitats.

A sum of 180 *I*. *granulatus* ticks, comprising 34 larvae, 83 nymphs, and 63 adults, were individually tested for presence of *Rickettsia*, with an overall prevalence of 8.3% (15/180). There was no difference in prevalence among larvae (5.9%, 2/34), nymphs (7.2%, 6/83), and adults (11.1%, 7/63) (*p* > 0.05). Prevalence was higher when ticks were collected from *M*. *musculus* (6.5%, 2/31), *R*. *losea* (12.3%, 7/57), and *S*. *murinus* (6.7%, 6/89) than from *R*. *norvegicus* (0%, 0/3) (all *p* < 0.05); there was no differences among the first three host species. Within *R*. *losea*, prevalence of *Rickettsia* presence in ticks was higher when *R*. *losea* was trapped in *L*. *leucocephala* invasion sites (15.6%, 7/45) than in agricultural fields (0%, 0/6), or human residential sites (0%, 0/6) (both *p* < 0.05). For ticks collected from *S*. *murinus*, prevalence was higher in invasion (7.8%, 5/64) and human residential sites (5.9%, 1/17) than in agricultural fields (0%, 0/8) (both *p* < 0.05), with no difference between the first two habitat types.

### Top soil temperature and relative humidity

**Fig 7:**
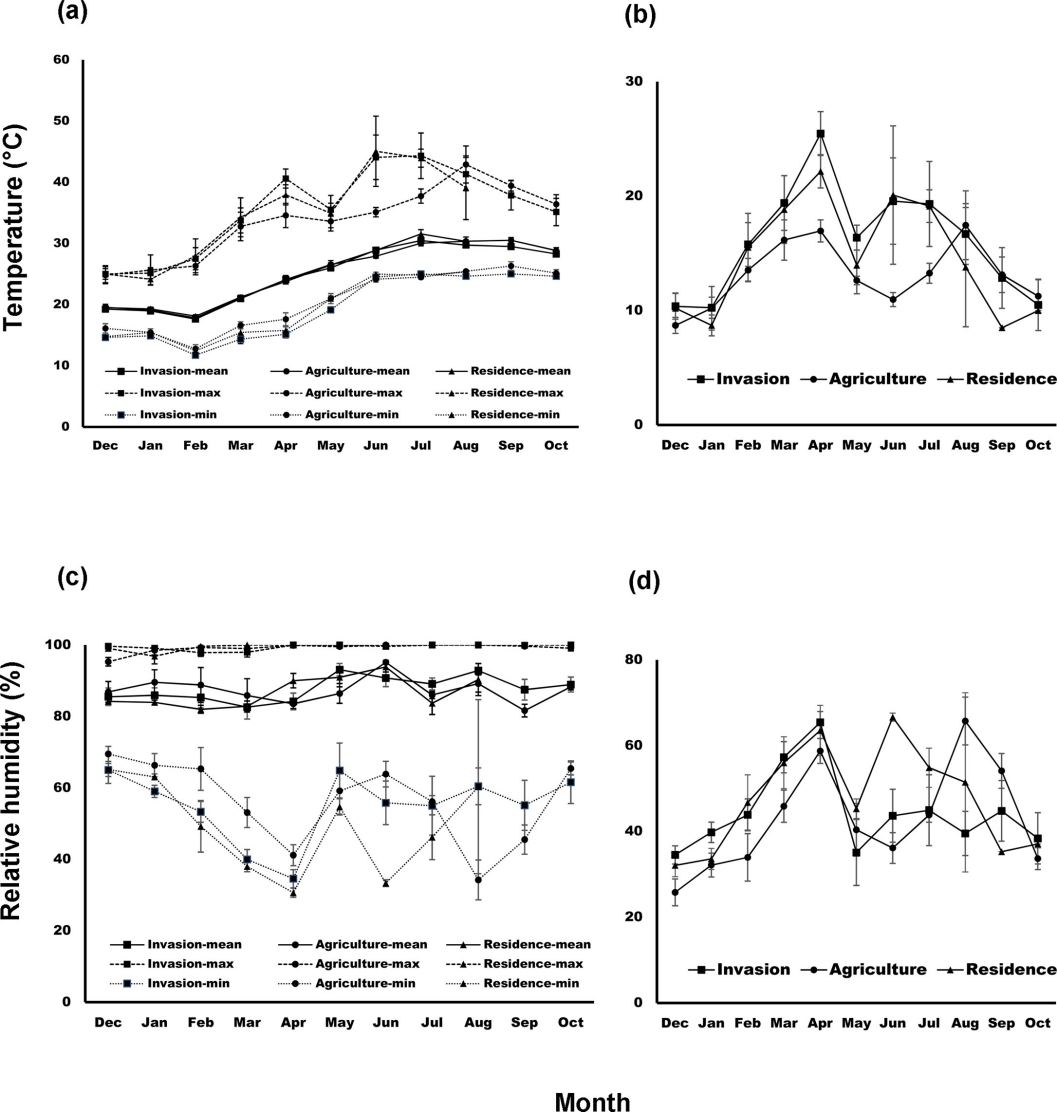
Monthly variation in temperature and relative humidity in different habitats in Penghu Islands from December 2016 to October 2017. (a) Minimum, mean, and maximum temperature; (b) fluctuation in monthly temperature (monthly maximum minus minimum); (c) minimum, mean, and maximum relative humidity; (d) fluctuation in monthly relative humidity (monthly maximum minus minimum).

There was no significant difference in the monthly mean, minimum, and maximum temperatures among the three habitats for each of the 11 months (Fig 7A, ANOVA, all *p* > 0.05). There was also no difference among habitat (all *p* > 0.05) in the variation in monthly temperature (monthly maximum minus minimum), except in April when invasion sites had higher variation than the agricultural fields (*p* < 0.05) (Fig 7B). In terms of relative humidity, there was no significant difference in monthly mean, minimum, maximum humidity, and variation in humidity among the three habitats for each of the 11 months (Fig 7C and 7D, ANOVA, all *p* > 0.05).

## Discussion

More chiggers and ticks infesting small mammals were collected from *L*. *leucocephala* invasion sites than from agricultural fields and human residential areas (Fig 6A and 6D). In addition, prevalence of OT in chiggers and *Rickettsia* in ticks was consistently not lower when vectors were collected from small mammals trapped in invasion sites compared to the other two habitats, demonstrating that the proliferation of invasive *L*. *leucocephala*, encouraged by abandonment of marginal agricultural fields after industrialization in Taiwan, has created hotspots for scrub typhus and potentially spotted fever. Moreover, *L*. *leucocephala* invasion sites maintained a significantly higher number of disease vectors during early winter (December) than the other two habitats (Fig 6A and 6D). We also found that the *R*. *losea* rodent and the *S*. *murinus* shrew were the primary hosts of chiggers and ticks, but engorgement degree of both vectors was much higher on *R*. *losea* than on *S*. *murinus* (Fig 4), suggesting that *R*. *losea* is a better host than *S*. *murinus*. Lastly, there was little difference in top soil temperature and moisture among the three habitats (Fig 7), but there were more *R*. *losea* in invasion sites (Fig 5A), suggesting that abundance of chiggers and ticks in invasion sites might be partially related to abundance of *R*. *losea* although it should be stressed that microclimate might not be well represented by only one measure point in each study site so its significance in determining abundance of vectors was not definitively solved and may still be worth investigating.

In this study, more than three quarters of chiggers were collected from *R*. *losea*, and degree of engorgement of chiggers from *R*. *losea* was 2.7-fold higher than those from *S*. *murinus*, suggesting that *R*. *losea* is the most important host of chiggers of the four species that we examined in Huxi Township of Penghu Islands. This result is in agreement with our previous large scale study that *R*. *losea* is the primary host of chiggers across Taiwan [82]. Furthermore, in contrast to other pathogens, such as *Borrelia burgdorferi*, in which transovarial transmission rarely occurs and vertebrate hosts are necessary for further infecting vectors [83], transovarial transmission of OT is high in chigger mites [33] and chigger mites are the only reservoir of OT [14] (although uninfected chiggers can acquire OT when feeding on infected hosts, the acquired OT is unable to be transmitted to the next generation [14]). Therefore, species identity of hosts will not affect transovarial transmission of feeding chiggers, that is, relative to *R*. *losea*, *S*. *murinus* will not be more critical in maintaining OT transmission. The observation that OT prevalence was greater in chiggers from *R*. *losea* than from other hosts might be related to the much higher chigger load, thus increased chance of encountering with infected chiggers of *R*. *losea*; this in turn elevates chance of chiggers infected with OT after feeding on more infective *R*. *losea*. A survey of shrews and rodents across Penghu Islands (instead of only Huxi) that included habitats of grasslands, fallow fields, agricultural fields, artificial facilities, and coastal windbreak plantations also found *M*. *musculus*, *R*. *losea*, and *S*. *murinus* to be the dominant species; *Rattus tanezumi* was also found but was in very low abundance and only occurred in one islet of Penghu Islands [84]. Therefore, *R*. *losea*, a species commonly occurs in habitats dominated by bush and grass in lowland Taiwan [85], is likely to be the most important host of chiggers not only in Huxi but across the Penghu Islands.

Although *L*. *deliense*, which is the primary chigger species vectoring OT in Southeast Asia [30], was also recognized as the dominant species in Penghu in this study as previous studies have shown [59,64], these past studies have instead identified *S*. *murinus* as the primary host of chiggers [58,59]. The reason for such inconsistency is unclear, particularly when both previous studies did not document in which habitat type the traps were set up. One possibility is that *L*. *leucocephala* was not widespread in the 1960s so there were much fewer *R*. *losea* at that time. On the other hand, our finding that *S*. *murinus* hosted >80% of chiggers specifically in human residential areas is similar to the result of [59], that 70% of chiggers were collected from *S*. *murinus*, suggesting that these past studies might have limited their survey to human residential areas. This current study, after including other habitat types, has instead uncovered *R*. *losea* as the most critical host of chiggers in Penghu. It should be noted, nevertheless, that more human activity surrounding human residential areas compared with *L*. *leucocephala* invasion sites suggests that chiggers active in human residential areas may be more pivotal in determining human risks to scrub typhus. In Penghu, *L*. *leucocephala* is so widespread that villages are typically surrounded by large tracts of this invasive plant. It thus warrants further investigation whether chiggers not well fed by *S*. *murinus* in human residential areas are required to be populated with chiggers well fed by *R*. *losea* from surrounding *L*. *leucocephala* invasion sites, similar to source-sink dynamics [86]. Additionally, whether preserving *S*. *murinus*, one of the most abundant commensal mammals in Taiwan (Fig 5C; [87]), can help lower chigger population size and thus human risks to scrub typhus.

Similar to previous studies (e.g. [3,7]), we investigated whether exotic plants have beneficial effects on disease vectors. Unlike other studies, however, we expanded upon conventional spatial comparisons to also track temporal vector population dynamics across seasons. We found that the abundance of chiggers on all small mammal hosts as well as an important host, *R*. *losea*, was not only higher in invasion sites, but seasonal fluctuation was also the lowest (with low CV), meaning that invasion sites have maintained a high and more stable chigger population during the study period. For example, during early winter (December), invasion sites still sustained a higher number of chiggers when the other two habitats had fewer chiggers, suggesting that *L*. *leucocephala* could be a temporary refuge for chiggers and helped prolong chigger survival under a less favorable climate. Likewise, abundance of ticks on *R*. *losea* was also higher and seasonally more stable in invasion sites. One exception though is that abundance of ticks on *S*. *murinus*, was also higher in invasion sites but displayed more dramatic seasonal fluctuation than human residential areas. This large fluctuation, however, was not due to low tick abundance during some months, but rather an exceptionally high tick abundance in August. Nevertheless, it should be emphasized that long-term research is warranted to assess whether the seasonal pattern we observed in a single year is consistent across multiple years.

The higher number of chiggers and ticks collected from small mammals in *L*. *leucocephala* invasion sites compared to the other two habitats is less likely due to the microclimate differences because soil temperature and moisture were similar among the three habitats. Instead, the difference in chigger abundance might be attributed to the higher number of *R*. *losea* found in invasion sites, which we also found to be a better host of chiggers although as stated above, microclimate might not be well characterized when only one data logger was placed in each study site. Invasive plants have been shown to help aggregate and increase rodent abundance by providing dense cover from predators [88]. Likewise, *R*. *losea* might be sheltered under the dense *L*. *leucocephala* cover, which in turn could increase chigger abundance. On the other hand, despite *R*. *losea* being the most critical host for chiggers, the relative contribution of *R*. *losea* and *S*. *murinus* to tick population was less clear. Compared to *R*. *losea*, which had the highest proportion of fully engorged ticks, *S*. *murinus* was an inferior host but was infested with more ticks. Because there were more *S*. *murinus* captured in human residential areas than outdoor fields (Fig 5C), as observed across Taiwan [87], the higher abundance of ticks in invasion sites may thus be unrelated to the abundance of *S*. *murinus*. Instead, *R*. *losea* might be crucial in sustaining the high tick population. Similar to chiggers, elucidating the survival of ticks on *S*. *murinus* is critical for assessing whether *S*. *murinus* act as sinks for ticks and if preserving *S*. *murinus* can help lower the risks of tick-borne diseases.

Density or abundance of questing, pathogen-infective ticks are considered a good indicator for human risks to tick-borne diseases and are commonly estimated with a dragging method (e.g. [89]). Moreover, free-living chiggers are typically sampled with black plate method [90]. However, the extremely dense *L*. *leucocephala* stands in our invasion sites have made the dragging method impossible. The black plate method was also not employed in this study due to very low collection efficiency in our preliminary study. Abundance of questing ticks and chiggers was therefore not directly quantified, but instead by quantifying abundance of infested vectors on the trapped hosts. It could be argued that more vectors on the hosts would mean fewer vectors left on the ground questing for humans. If this is true, however, ectoparasite loads on hosts will be lower after the first cohort of emerged vectors finished their meal on the hosts and return to soils to molt or lay eggs. High abundance of chiggers and ticks on the hosts should thus reflect a continuous supply of questing chiggers and ticks in that habitat although whether the magnitude of difference in vector abundance observed on hosts realistically reflects differences in questing vectors requires further investigation. The other limitation of this study is that throughout the Penghu Islands, *L*. *leucocephala* is so widespread that we were unable to include sites inhabited solely by native plants for comparison. However, unlike native plants, eradicating *L*. *leucocephala* is extremely difficult due to its large soil seed bank (around 2,000 seeds per square meter [57]). Eradicating *L*. *leucocephala* for controlling vector-borne diseases will therefore be more challenging than removing native plants even when the latter habitat also contains many disease vectors. From a public health perspective, investigating whether invasion sites are hotspots of vector-borne diseases warrants more concern. In addition, it is possible that the environment conducive to *L*. *leucocephala* invasions happen to also be favorable for the survival of chiggers and ticks. Manipulative studies that remove *L*. *leucocephala* and compare chigger and tick abundance before and after the removal should help assess the relative importance of this invasive species in harboring both vectors. A comparison between *L*. *leucocephala*-removal sites that have been naturally colonized with the invasive species and sites where native shrub species have been introduced could help evaluate the real significance of exotic plants instead of plant structure (e.g. plant density and height) in facilitating disease vectors. Lastly, it is plausible that habitat size can have scale-dependent effects on the abundance of hosts and vectors so that there might be fewer hosts and vectors in the much smaller agricultural fields than *L*. *leucocephala* invasion and human residential sites. We did not observe such an effect because there was no difference in the number of hosts trapped among the three habitats. In addition, abundance of infested vectors was higher (for chiggers) or similar (ticks) when small mammal hosts were trapped from agricultural fields relative to human residential sites (Fig 6), suggesting that habitat characteristics instead of habitat size are more important in determining abundance of hosts and vectors.

This study highlights an important but largely neglected issue that a change in socioeconomics, such as a shift from agriculture to service and industry sectors, will stimulate a dramatic alteration on land use, which in turn can have considerable consequence for human risks to vector-borne diseases. The change in vegetative community composition accompanied with land use conversion, particularly invasions of exotic plants, will not only interfere with the recovery of native fauna and flora but can potentially provide refuges for disease vectors and their vertebrate hosts even under unfavorable weather, hence increasing disease risks for the general public.

## Supporting information

S1 Fig Number of vectors collected from mammal hosts per study site in different regions of Penghu Islands from December 2016 to October 2017. (DOCX)
