# Supplementary material for: Invasive plants facilitated by socioeconomic change harbor vectors of scrub typhus and spotted fever
Source: PLoS Negl Trop Dis. 2020 Jan 21;14(1):e0007519. doi: 10.1371/journal.pntd.0007519 (PMC6994164; doi:10.1371/journal.pntd.0007519)

Fig. S1. Number of vectors collected from mammal hosts per study site in different regions of Penghu Islands from December 2016 to October 2017. Chiggers collected from (a) all mammals combined; (b) *Rattus losea*; (c) *Suncus murinus*. Ticks collected from (d) all mammals combined; (e) *Rattus losea*; (f) *Suncus murinus*. Different letters represent significant difference. Error bar +1SE.


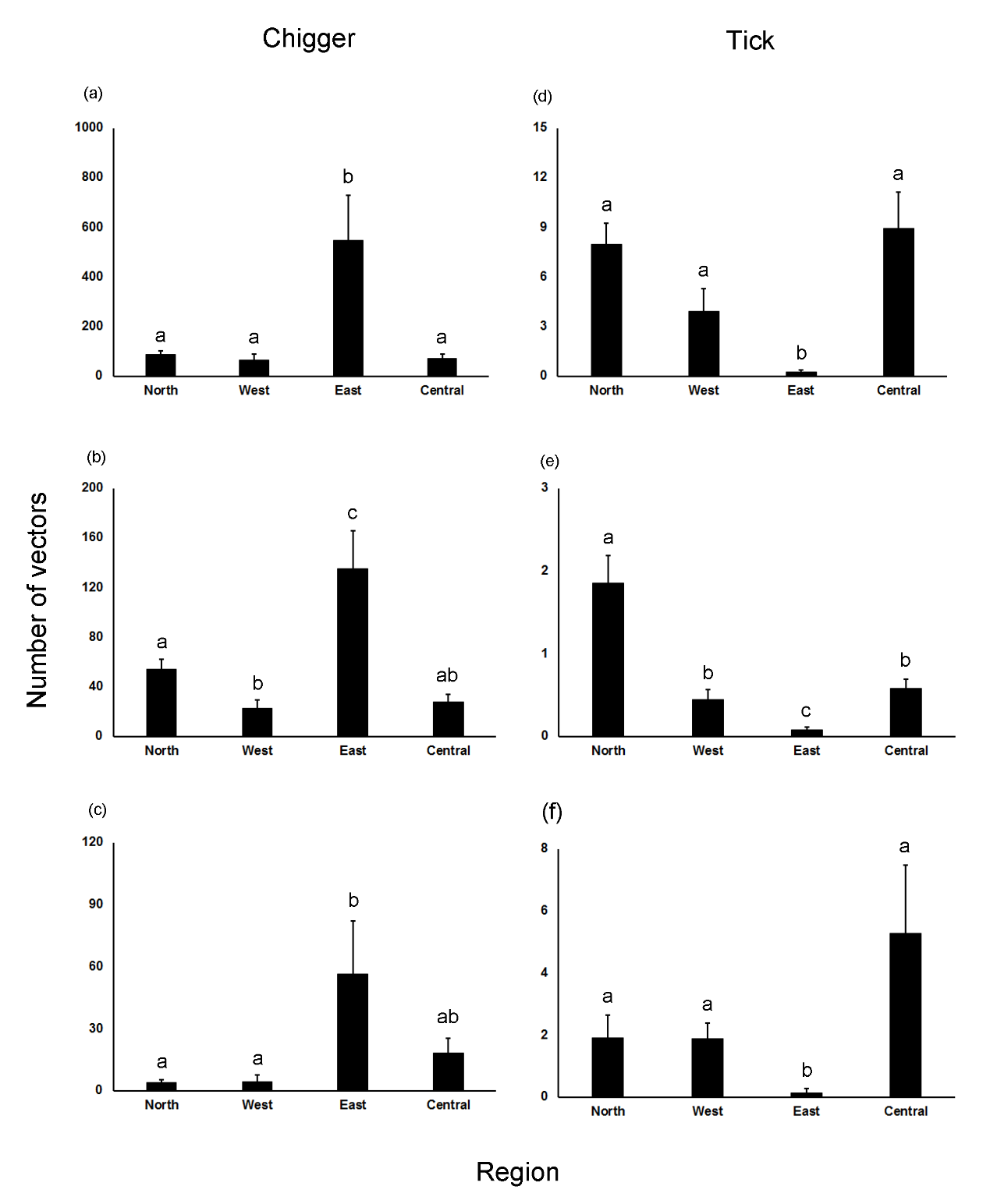

Supplement: S1 Fig — (DOCX) [file pntd.0007519.s001.docx]
